# Nicotinic Acetylcholine Receptors in the Respiratory Tract

**DOI:** 10.3390/molecules26206097

**Published:** 2021-10-09

**Authors:** Monika I. Hollenhorst, Gabriela Krasteva-Christ

**Affiliations:** Institute of Anatomy and Cell Biology, Saarland University, 66424 Homburg, Germany; gabriela.krasteva-christ@uks.eu

**Keywords:** nicotinic acetylcholine receptors, airways, lung, respiratory tract, mucociliary clearance, chronic obstructive pulmonary disease, asthma, lung cancer

## Abstract

Nicotinic acetylcholine receptors (nAChR) are widely distributed in neuronal and non-neuronal tissues, where they play diverse physiological roles. In this review, we highlight the recent findings regarding the role of nAChR in the respiratory tract with a special focus on the involvement of nAChR in the regulation of multiple processes in health and disease. We discuss the role of nAChR in mucociliary clearance, inflammation, and infection and in airway diseases such as asthma, chronic obstructive pulmonary disease, and cancer. The subtype diversity of nAChR enables differential regulation, making them a suitable pharmaceutical target in many diseases. The stimulation of the α3β4 nAChR could be beneficial in diseases accompanied by impaired mucociliary clearance, and the anti-inflammatory effect due to an α7 nAChR stimulation could alleviate symptoms in diseases with chronic inflammation such as chronic obstructive pulmonary disease and asthma, while the inhibition of the α5 nAChR could potentially be applied in non-small cell lung cancer treatment. However, while clinical studies targeting nAChR in the airways are still lacking, we suggest that more detailed research into this topic and possible pharmaceutical applications could represent a valuable tool to alleviate the symptoms of diverse airway diseases.

## 1. Introduction

Nicotinic acetylcholine receptors (nAChR) are widely distributed in diverse neuronal and non-neuronal tissues. They form various subtypes consisting of different subunit combinations. In mammals, nine alpha subunits (α1-7, α9, α10), four beta subunits (β1-4), and one delta (δ), gamma (γ) and epsilon subunit (ε) have been detected [1]. The α1, β1, δ, γ, and ε subunits are restricted to the muscle [1]. The nAChR form predominately ionotropic pentameric receptors permeable for Ca^2+^ and Na^+^, albeit with different affinities depending on the nAChR subtype [1]. However, there is a notion that nAChR can also have metabotropic functions, especially in immune cells. For example, in T cells, the α7 nAChR increase the intracellular Ca^2+^ level via protein kinase activation instead of being permeable to Ca^2+^ [2].

In the respiratory tract, various subunits of nAChR have been detected. In rat tracheal epithelium, all mammalian α subunits but α1 have been identified [3]. In mouse tracheal epithelium, we have been able to discover the α3, α4, α5, α7, α9, α10, β2, and β4 subunits, with the α3 and α10 subunits being the most abundant among all of the α subunits, followed by the α4 and α7 subunits [4,5]. In an α7 reporter mouse strain, expression of the α7 nAChR was specifically detected in the epithelial club cells and alveolar type II cells [6]. In the mouse embryonic lung, the α7 as well as the α5 subunits play an important role in lung development, as indicated by the strict regulation of transcription of both subunits during lung morphogenesis [7,8]. In bronchial epithelial cells of rhesus macaques, the α3, α4, α7, α9, α10, β2, and β4 subunits have been detected [9]. Surprisingly, various nAChR subunits have also been found in human bronchial epithelial cells, namely the α3, α5, α7, β2 and β4 subunits [10,11]. Additionally, a recent study detected almost all of the nAChR subunits, with the exception of α7, in adult human whole-lung samples from non-smokers, which contained all types of tissues, including epithelia, muscle, connective, and nervous tissue [12]. When the authors analyzed the epithelial samples of the large airways, they found all of the subunits except α1, α2, α4, β1, β3, β4, and δ, whereas in the small airways, they discovered all 16 subunits. Remarkably, the authors found differences in the nAChR subunit expression between smokers and non-smokers, such as the expression of the α7 subunit in whole-lung samples. The expression of α5, α7, α10, β2, and β3 was mainly correlated with smoking. In addition to peripheral lung tissue and the airway epithelium, nAChR are also present on the immune cells and nerve endings innervating the airways [2,13]. Interestingly, one study also described the intracellular localization of nAChR in the lung tissue in the outer mitochondrial membrane [14]. The authors were able to detect the α3, α4, α7, β2, and β4 subunits with a sandwich assay using subtype specific antibodies, with the α3 and β4 subunits being the most abundant in the lung. Though these findings are very intriguing, in light of the well-recognized problems regarding the specificity of the available antibodies for the detection of AChR [15,16], these findings should be verified in terms of this aspect, including transgenic mice (knock-out, reporter mouse strains or/and overexpressing mouse strains) other than α7 and β2 nAChR-deficient mice. Nevertheless, studies that have been performed with keratinocytes have provided clear evidence that α3, α5, α7, α9, α10, β2, and β4 nAChR are functional mitochondrial proteins [17]. Taken together, all of the aforementioned studies show that while some differences might exist between species in terms of the expression of the various nAChR subunits, they seem to be broadly expressed and to play an essential role for the functions of the respiratory tract. This is further underlined by a study that showed a correlation between nAChR expression and lung function measured by FEV1 [18].

Generally, nAChR display a wide variety of effects and properties. This is based on the multitude of subunits present in different cell types in the respiratory tract, which results in various possibilities for the assembly of these subunits. For example, the α7 and α9 subunits are able to assemble as homopentamers or to form functional heteropentamers, such as α9α10 or α7β2 [19,20]. Additionally, heteropentameric receptors can adopt various stoichiometries. The α7β2 nAChR may contain one, two, or three β subunits but needs at least two α subunits to form functional receptors [19]. Other receptors can contain two α subunits and three β subunits or three α subunits and two β subunits, as was shown for the α3β4 nAChR expressed in HEK293 cells and in *Xenopus* oocytes as well as for the α4β2 nAChR expressed in *Xenopus* oocytes [21,22]. These variants in the stoichiometries result in different affinities to ACh for the α3β4 as well as the α4β2 nAChR [21,22] and may have different pharmacological profiles, as shown for the α7β2 nAChR [19]. Recently, a stoichiometry of 3α:2β subunits was determined for the nAChR in synapses between motoneurons and Renshaw cells [23]. Additionally, nAChR properties can be modulated by assembly with the α5 nAChR subunit to form, e.g., α3β4α5 or α4β2α5 nAChR, as recently reviewed [24].

Acetylcholine (ACh) serves as an endogenous ligand for the nAChR. In the airways, ACh may originate from two different sources: (a) neuronally from the innervating cholinergic nerve endings [25] or (b) non-neuronally from cholinergic chemosensory tracheal brush cells, which represent a rare epithelial cell type expressing the ACh synthesizing enzyme choline acetyltransferase [26,27,28]. Additionally, nose solitary chemosensory cells are cholinergic and express choline acetyltransferase [29]. The nAChR subtypes display diverse physiological properties, as discussed in this review, since their function depends on the subunit composition and on their affinity to their endogenous ligand ACh.

## 2. The Role of Nicotinic Acetylcholine Receptors in Mucociliary Clearance

In the airways, mucociliary clearance (MCC) is an essential innate immune process. It serves to transport inhaled particles and pathogens out of the airways by a continuous, orchestrated ciliary beat, thereby preventing infections. MCC is an evolutionarily conserved mechanism that can be found throughout the animal kingdom and that is already present in corals [30]. In the airways, it consists of two components: a physical component resulting from the ciliary beat of the ciliated epithelial cells and a chemical component defined by the volume and viscosity of the airway surface liquid (ASL). The viscosity and height of the ASL is mainly regulated by ion transport processes. The ASL is, in turn, composed of the periciliary liquid surrounding the cilia and providing an optimal environment for the beating of the cilia, which is covered by a mucus layer in which the inhaled particles and pathogens are trapped and then transported out of the airways together with the mucus.

It has been known for a while that ACh is able to regulate mucociliary clearance by influencing ciliary beat and ion transport processes. ACh and the agonist of the muscarinic acetylcholine receptors, muscarine, have been shown to influence particle transport speed in mouse tracheas, mainly via the M3 receptors [27,31]. The cholinergic action on ciliary beat depends on intracellular Ca^2+^, protein kinase A, and protein kinase G [32,33]. Interestingly, ACh is also able to regulate transepithelial ion transport processes, which was demonstrated about 25 years ago in sheep trachea [34]. However, at that time, ACh was thought to originate solely from neurons. With the idea that a non-neuronal cholinergic system might also exist in the respiratory tract, ACh of a non-neuronal origin was shown to be involved in the regulation of transepithelial ion transport, as investigated in mouse and pig tracheal epithelium [35,36]. Not only muscarinic receptors but also nAChR are involved in regulating mucociliary clearance. We have previously shown that in murine tracheal epithelium, the activation of nAChR with nicotine opens apical Cl^−^ and basolateral K^+^ channels, more precisely, TMEM16A and KCNQ1 via Ca^2+^ and cAMP dependent pathways downstream of the α3β4 nAChR [4,5,37]. These studies point towards a metabotropic rather than an ionotropic action of the nAChR, providing further evidence that the classical picture of nAChR being ligand-gated ion channels needs to be revised. Recently, nAChR have also been shown to influence the ciliary-mediated component of mucociliary clearance by increasing cilia driven particle transport in murine tracheas via the same nAChR subtype, namely α3β4, which is responsible for mediating the changes in ion transport processes [38]. These studies about nAChR and mucociliary clearance have been conducted in mice. In the pig trachea, which is supposed to be a more relevant model with regard to the transfer of results to humans, nicotine had no effect on tracheal epithelial ion transport [36]. Therefore, one could argue that there might be no relevance for humans. However, a study performed before the existence of a non-neuronal cholinergic system in the airways became widely apparent showed that nicotine influences transepithelial ion transport and intracellular Ca^2+^ levels in human nasal epithelial cells [39]. This provided the first evidence of functional nAChR in non-excitable cells. Additionally, the α7 nAChR is able to regulate ion channels, mainly the cystic fibrosis transmembrane conductance regulator (CFTR) channel in human airway epithelium [40]. Thus, in the case of studying the role of nAChR in mucociliary clearance, the mouse seems to be a more relevant model for extrapolating the results to humans than pig models are. However, of course, more detailed studies on the role of nAChR in mucociliary clearance in humans are needed. Taken together, the studies in mouse and human airway epithelium regarding the regulation of mucociliary clearance by nAChR point towards nAChR, especially the α3β4 subtype, being a suitable therapeutic target for the stimulation of the mucociliary clearance under disease conditions in which it is impaired, such as in cystic fibrosis and in chronic obstructive pulmonary disease (COPD).

## 3. Nicotinic Acetylcholine Receptors in Asthma and Chronic Obstructive Pulmonary Disease

Asthma and COPD are diseases of the respiratory tract, both of which are characterized by an obstruction of the airways. The cholinergic system is of considerable interest in these diseases since it is involved in bronchoconstriction, which is mediated by ACh acting mainly at the muscarinic M3 receptor [41]. Taking this into consideration, it is not surprising that anticholinergic therapy for muscarinic receptors has been discussed for COPD as well as in asthma [42,43]. However, this is only part of the story, as these diseases are not only characterized by airway obstruction but also by inflammation. A role for cholinergic signaling for inflammatory processes has been well established for a mechanism called the cholinergic anti-inflammatory pathway. In this model, stimulation of the vagus nerve inhibits inflammatory responses. Twenty years ago, the α7 nAChR had already been identified as playing a critical role in the cholinergic anti-inflammatory pathway and as being involved in anti-inflammatory effects [44]. A recent study revealed that α7 nAChR acts via an activation of adenylyl cyclase 6 and a lipid raft-mediated endocytosis of TLR4 in a COPD mouse model and proposed the use of α7 agonists as a novel approach for COPD treatment [45]. In line with this, another study revealed that IL6 and NO levels were increased in the blood plasma of COPD patients with peripheral blood mononuclear cells and that these levels were associated with impaired lung function. Both, IL6 and NO were downregulated when patients were treated with GTS-21, an α7 nAChR agonist [46], underlining the importance of nAChR-mediated anti-inflammatory action also for COPD in humans. In a recent study, the same agonist was proven to be beneficial in a mouse model of hyperoxia-induced acute inflammatory lung injury [47]. There, the activation of the α7 nAChR reduced the levels of high-mobility group box 1 (HMGB1), a protein secreted by macrophages, monocytes, and dendritic cells in the airways and in the circulation. Another hallmark of COPD is the dysfunction of airway smooth muscle cells, which contributes to airway remodeling and obstruction [48]. In this context, it should be considered that nAChR also play a role in airway smooth muscle proliferation. It has recently been shown that nicotine-induced proliferation of airway smooth muscle cells is mediated by α7 nAChR since α7 nAChR-specific siRNA and inhibition with the α7 nAChR antagonist MG624 attenuated proliferation [49]. The α7 nAChR acted via a mechanism that involves transient receptor potential (TRP) C6 channels and PI3K/Akt signaling [49]. Another study found that the α5 nAChR mediates airway smooth muscle cell proliferation via TRPC3 channels [50]. Thus, targeting nAChR and, in particular, the α7 nAChR in COPD needs to be controlled carefully. On the one hand, it might have beneficial effects by attenuating inflammation, and on the other hand, it might have deteriorative effects by increasing airway smooth muscle proliferation. Nevertheless, a combined therapy for activation of α7 nAChR to reduce inflammation and inhibition of α5 nAChR or TRPC3 channels to reduce airway smooth muscle cell proliferation might represent a promising therapeutic approach for COPD.

Similar to COPD, α7 nAChR-dependent signaling seems to play a role in asthma. In an ovalbumin asthma mouse model, the membrane impermeable nAChR agonist 1,1-dimethyl-4-phenylpiperazinium (DMPP) was able to attenuate the airway hyperresponsiveness, a hallmark of asthma, and airway inflammation by reducing lymphocyte and eosinophil numbers in the bronchoalveolar lavage fluid and tissue infiltration by mononuclear cells and eosinophils [51]. Moreover, the eosinophilia was suppressed by normal α7 nAChR signaling in wild-type mice compared to in mice with a mutated dysfunctional α7 nAChR in asthma models with house dust mites or ovalbumin [52].

Besides eosinophilia, group 2 innate lymphoid cells (ILC2) play an important role in asthma and allergic diseases in generating type 2 immune responses, as recently reviewed by Zheng and coworkers [53]. A recent study discovered that vagotomy reduced the number of ILC2 in an ovalbumin-induced asthma model of allergic inflammation and that this effect was dependent on α7 nAChR, as ILC2 numbers were increased in α7 nAChR-deficient mice [54]. Additionally, the application of an α7 nAChR agonist resulted in reduced cytokine expression in ILC2, and attenuated ILC2-induced airway hyperreactivity [55]. In line with this, targeting the α7 nAChR with the specific agonists PNU-282987 or GTS-21 reduced the ILC2 numbers in the bronchoalveolar lavage fluid, goblet cell hyperplasia, and the eosinophilic infiltration and thus attenuated airway inflammation in mice treated with IL33 or the fungus *Alternaria alternata* to induce inflammation [56] Moreover, type 2 immune responses can also be elicited by tracheal epithelial brush cells releasing ACh upon activation with bitter taste receptor agonists or with bacterial substances [57]. However, the role of brush cells in asthma and COPD in humans remains elusive.

## 4. Additional Functions of Nicotinic Receptors in the Respiratory Tract

Besides their role in mucociliary clearance, COPD, asthma, inflammation, infection, and lung cancers, nAChR play other important roles in the physiology and pathophysiology of the respiratory system. A rat model of ventilator-induced lung injury revealed that the activation of the α7 nAChR with the agonist PNU-282987 prior to ventilation attenuated the ventilator-induced injury [58]. In line with this, the activation of the cholinergic anti-inflammatory pathway and especially of the α7 nAChR subunit has been previously suggested as an alternative treatment for ventilator-induced lung injury [59]. Not only in ventilator-induced lung injury but also in a model of acid-induced acute lung injury, the stimulation of the α7 nAChR had beneficial effects by attenuating this type of lung injury, as indicated by decreased pulmonary edema, reduced vascular permeability, and a lower protein component in the bronchoalveolar lavage fluid [60]. The expression of collagen type I in cultured primary mouse lung fibroblasts was found to be upregulated via α7 nAChR stimulation in vitro [61]. In vivo treatment with nicotine increased the collagen type I expression in wild-type but not in α7 nAChR-deficient mice, which points towards the involvement of the α7 nAChR subunit in the inflammatory extracellular matrix remodeling process in response to lung injury [61].

Previously, we have been able to show that nerve endings approaching the airway epithelium and especially the chemosensory cholinergic brush cells in the airways also express nAChR and, specifically, the α3 nAChR subunit [26]. In line with this, we were able to show that the stimulation of taste 2 receptors in the airways by the bitter substance denatonium led to a transient decrease in the respiratory rate and induced respiratory events similar to cough reflexes in humans in a nAChR-dependent manner [27]. Cough and defensive airway reflexes have been shown to be dependent on nAChR [62]. However, the role of brush cells for nAChR-dependent nerve activation in humans needs to be elucidated.

## 5. The Role of Nicotinic Acetylcholine Receptors in Monocytes and Macrophages

In addition to the presence of α7 nAChR in ILC2 cells, nAChR are also expressed in monocytes and macrophages, both of which are immune cell types that are widely present in the respiratory tract and particularly in the alveolar region. Alveolar macrophages play a key role in the innate immune response elicited by inhaled pathogens or particles. In rat alveolar macrophages, several subunits of nAChR have been detected, such as the α3, α5, α9, α10, β1, and β2 subunits. Therefore, diverse functional subtypes may exist. However, in these cells, no nAChR-dependent membrane currents or an increase in intracellular Ca^2+^ levels due to nicotine or ACh application could be detected, pointing towards a metabotropic action of nAChR in alveolar macrophages [13]. Indeed, an attenuation of an increase of the intracellular Ca^2+^ concentration due to purinergic signaling could be observed in this study when the nAChR in the alveolar macrophages were activated by nicotine prior to stimulation with ATP, indicating that the nAChR on alveolar macrophages are functional while not forming ligand-gated ion channels themselves. The assumption that the nAChR in the macrophages are metabotropic is supported by a study with peritoneal macrophages, where the α7 nAChR was activated by the Janus kinase 2 pathway [63]. Moreover, the expression of the α7 nAChR in mouse alveolar macrophages was clearly shown by observing fluorescence in a tau-GFP reporter mouse strain (α7^G^), in which a bicistronic IRES:tau-GFP expression cassette was added to the 3′ end of the α7 nAChR transcript [6]. The α7 nAChR in the alveolar macrophages seem to play an important role in acute lung injury. In a lipopolysaccharide (LPS)-induced model of acute lung injury, the α7 nAChR agonist GTS-21 was able to prevent the lung injury and to decrease the number of alveolar macrophages and their production of pro-inflammatory cytokines [64]. Similar results were obtained using the α7 nAChR agonist PNU-282987 [65]. Strikingly, not only do the α7 nAChR on the macrophages play an anti-inflammatory role but the α7 nAChR in the airway epithelium are also involved in cytokine and chemokine production after LPS challenge [6]. Taken together, this clearly shows the importance of the role of α7 nAChR on immune cells.

The expression of the α7 nAChR has been shown for peripheral blood mononuclear cells [44]. A population of migrating monocytes has been identified in the lung [66]. The expression of α7 nAChR was detected in the human monocyte cell line THP-I and in monocytes from smokers [67]. However, the α7 nAChR is not the only nAChR subtype that can be found in monocytes. A metabotropic role was detected for the α9 and α10 subunit containing nAChR in mouse monocytes [68]. Upon activation with phosphocholine, these receptors inhibited the P2X7 ATP receptors, which resulted in a repression of the ATP-dependent release of the pro-inflammatory cytokine interleukin-1β (IL-1β) and thus in an ATP-dependent inflammasome activation. The effect of nAChR on the ATP-induced release of IL-1β has been elucidated in detail by Grau and co-workers and is described in the following paragraph [69,70,71,72,73,74]. The authors discovered that the monocytic α7, α9, and α10 nAChR subunits inhibited the IL-1β release when activated by phosphocholine, lysophosphocholine, and glycerophosphocholine. The α7 nAChR subunit was only marginally involved in this metabotropic effect [69]. This effect seems to act by a feedback loop since the C-reactive protein, which is produced in in response to increased IL-1β levels and binds to phosphocholine, inhibited the ATP-induced IL-1β release via nAChR [70]. Additionally, alpha-1 antitrypsin, a serine-protease that protects the human body from excess enzyme activity in inflammation, was able to inhibit ATP-dependent IL-1β release in monocytes via a mechanism that involves nAChR [71]. Moreover, this phenomenon is not only relevant for monocytes but is also relevant for the respiratory tract [72]. Phosphocholine and phosphocholine-modified lipooligosaccharides from *Haemophilus influenzae* were able to inhibit IL-1β release in the lung adenocarcinoma epithelial cell lines A549 and Calu-3 as well as in precision-cut lung slices [72]. Monocytes in the lung are constantly exposed to surfactant. Interestingly, the surfactant component dipalmitoylphosphatidylcholine was able to inhibit ATP-dependent IL-1β release via nAChR in monocytes [73].Thus, this component could represent an important endogenous regulator of inflammation. In contrast to this, the amyloid beta peptide Aβ1-42 overrode the anti-inflammatory effects of nAChR in monocytes [74]. Taken together, nAChR-dependent inflammasome inhibition creates further evidence for the importance of the anti-inflammatory action of nAChR and renders the α9α10 nAChR in monocytes and the respiratory tract a suitable pharmaceutical target for the inhibition of excessive inflammatory response.

## 6. The Role of Nicotinic Acetylcholine Receptors in Infections

Given the presence of nAChR in monocytes and macrophages and their ability to influence immune responses, it is not surprising that nAChR also play a role in infectious diseases. In lung epithelial cells and macrophages infected with *Mycobacterium tuberculosis,* nicotine treatment led to a reduction in several cytokines and chemokines such as IL-6, IL-8, IL-10, tumor necrosis factor α, C-C chemokine ligand (CCL) 2, CCL5, and C-X-C chemokine ligand (CXCL) 9 or CXCL10 in an nAChR-dependent manner [75]. Consistent with this, an inhibitory action of the α7 nAChR on cytokine production after Toll-like receptor activation in human monocytes was previously described [76]. In addition to these anti-inflammatory effects on the cytokines, an inhibitory effect of nicotine on the expression of antimicrobial peptides in type II pneumocytes and airway basal epithelial cells could also be observed when these cells were infected with *M. tuberculosis* [77]. This resulted in the increased growth of *M. tuberculosis* in type II pneumocytes [77]. However, while promoting the anti-inflammatory effects of nAChR by targeting them pharmaceutically might be beneficial in airway diseases with excessive inflammation in the stage before bacterial infection, such as acute lung injury, COPD, asthma, and even cystic fibrosis, targeting nAChR in infectious diseases where cytokine production and the production of antimicrobial peptides is crucial might be disadvantageous.

Since last year, the role of nicotine in COVID-19 has been controversially discussed. A study from Lupacchini and co-workers concluded that nicotine aggravates SARS-CoV-2 infections [78]. This assumption is based on the increased proliferative effect of nicotine and a nAChR-dependent upregulation of ACE2, as ACE2 has previously been shown to play a role in COVID-19 infection [79]. However, experiments with SARS-CoV-2 infection in combination with nicotine need to be performed in future studies in order to better support the conclusion of the authors. In contrast, Miyara and co-workers found a lower proportion of smokers amongst COVID-19 patients than in the general population [80]. This study has strengths because it is based on direct interviews with patients and by separating inpatients and outpatients. However, it also has limitations. It is a single-center study, and the authors excluded patients in intensive care units [80]. Nevertheless, a recent review has suggested the nicotinic cholinergic system as a possible therapeutic target based on the anti-inflammatory properties of nicotine to counteract the cytokine storm observed in severe COVID-19 cases and on its neuroprotectant and mood improvement effects [81]. Since the role of nicotine in COVID-19 disease has recently been reviewed elsewhere [81,82], it will not be discussed further and in more detail in the present review article. Thus, with the recent emergence of SARS-CoV-2 virus resulting in a severe pandemic, it has become evident that nAChR also play a role in viral infectious diseases.

## 7. The Role of Nicotinic Acetylcholine Receptors in Lung Cancer

So far, the effects of nAChR and especially of the α7 nAChR on cell proliferation and their role in lung cancer have been extensively studied and recently reviewed in detail [83]. Therefore, this paragraph aims to highlight only a few key findings regarding the role of nAChR in lung cancer. In recent years, a specific role of the α7 nAChR in lung cancer emerged since α7 nAChR mediated cell proliferation via a mechanism that involved β-arrestin-dependent signaling with a downstream Src activation and an interaction with Rb-Raf-1, which is elevated in non-small cell lung cancers [84]. The activation of this metabotropic α7 nAChR signaling pathway resulted in the increased proliferation of non-small cell lung cancer cells due to mitogenic effects on these cells [84]. Another study suggested that the α7 nAChR gene was upregulated via the transcription factors E2F1 and STAT1 when non-small cell lung cancer cells were stimulated with nicotine [85]. Furthermore, an α7 nAChR-dependent activation of the MEK/ERK signaling pathway was found to play a role in non-small cell lung cancer progression, especially during epithelial–mesenchymal transition, in tumor growth, and in vimentin expression [86,87]. In conclusion, inhibiting the α7 nAChR and its various pathways that are involved in lung cancer seems to be a promising strategy to combat lung cancer.

In addition to α7 nAChR, α9 nAChR are also involved in lung cancer and promote nicotine-induced proliferation in the A549 lung adenocarcinoma cell line via Akt- and ERK-dependent pathways [88]. In the bronchial epithelial cell line BEPD2, different variants of the α9 nAChR have been identified due to naturally occurring polymorphisms [89]. An analysis of the function of these isoforms by overexpression revealed that one variant (full length S442 protein) increased cell proliferation and transformation, while another variant (full length N442 protein) did not influence the proliferation but reduced the transformation of the human bronchial epithelial cell line BEP2D. A truncated form of α9 nAChR decreased proliferation as well as transformation [89]. This indicates that α9 nAChR polymorphism is an important factor determining increased susceptibility to lung cancer development. Furthermore, the gene cluster encoding the α5, α3, and β4 nAChR subunits plays an important role in lung cancer [90]. In non-small cell lung cancer, the activation of the α5 nAChR induces the expression of the hypoxia-inducible factor 1α and the vascular endothelial growth factor via the ERK1/2 and PI3K/Akt pathways involved in tumor cell proliferation [91]. Supportively, the α5 nAChR and the hypoxia-inducible factor 1α were both upregulated in non-small cell lung cancer cells [91]. In the lung adenoma cell line A549, α5 nAChR have been shown to activate cell migration and cell invasion [92]. In mice injected with A549 cells stably suppressing α5 nAChR, tumor growth development was reduced upon α5 nAChR suppression [93]. As a downstream mechanism for α5 nAChR in nicotine-induced lung cancer, JAK2/STAT3 has been identified [94]. More recently it was also shown that Jab1/Csn5 expression was correlated with α5 nAChR expression in lung cancer and that it increased the expression of N-cadherin and vimentin, which is indicative for an induction of epithelial-mesenchymal transition [95]. Taken together, these studies show that inhibiting α5 nAChR might represent a promising target for therapy for nicotine-induced non-small cell lung cancer.

Interestingly, the subcellular localization of nAChR also seems to play a role in the effects of nAChR on lung cancer progression. Chernyavsky and coworkers [96] found that cytoplasmic membrane nAChR had a growth-promoting effect on lung cancer tumors by synergizing with growth factors, while mitochondrial nAChR were involved in the anti-apoptotic action of nicotine, further contributing to tumor progression.

## 8. Metabotropic Signaling of Nicotinic Acetylcholine Receptors

**Figure 1:**
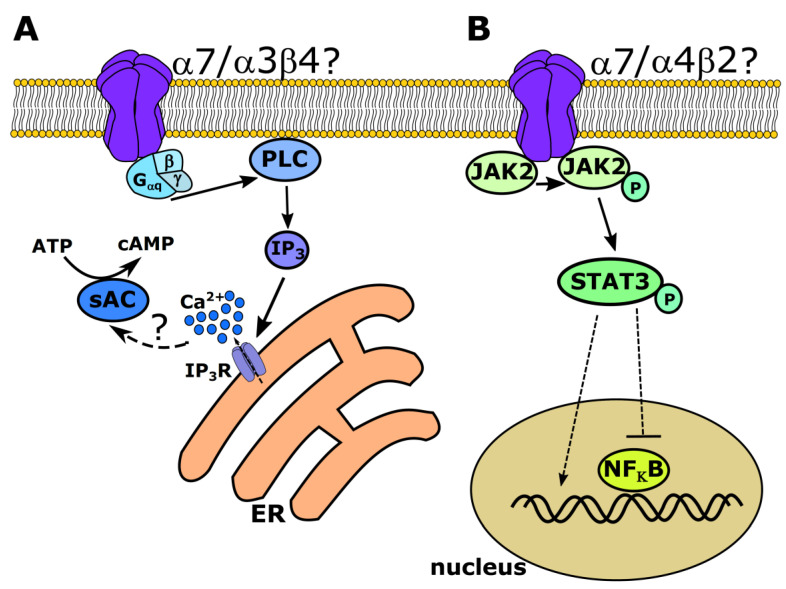
Examples of possible metabotropic signaling pathways for nicotinic acetylcholine receptors (nAChR). (**A**) The α7 nAChR have been shown to couple to G_αq_ proteins, which then activates phospholipase C_β2_ (PLC), resulting in an inositol 1,4,5-trisphosphate (IP_3_)-dependent release of Ca^2+^ from intracellular stores. The α3β4 nAChR in the airway epithelium have also been shown to act via a metabotropic IP_3_-dependent release of intracellular Ca^2+^ by a yet uninvestigated pathway, which might then lead to the activation of Ca^2+^-dependent soluble adenylyl cyclases (sAC), increasing intracellular cAMP. (**B**) The α7 nAChR may also be coupled to Janus kinase 2 (JAK2) and may activate the signal transducer and activator of transcription 3 (STAT3), which then either regulates gene expression itself or inhibits NF_κ_B. Additionally, for α4β2 receptors, JAK2/STAT3-dependent signaling has been described but not fully elucidated.

Often, the different properties of the nAChR resulting from variable subunit composition and stoichiometry have been characterized based on ionotropic receptor functions. However, this is only part of the story, as there is increasing evidence for metabotropic signaling in nAChR as an important and interesting new area of research, as described above. However, the exact mechanisms of metabotropic signaling, especially in the respiratory tract, are not fully elucidated. Several mechanisms of metabotropic signaling have been attributed to α7 nAChR. In the brain, α7 nAChR have been shown to form complexes with G proteins [97]. The activation of these receptors induces a G_αq_-, phospholipase C_β2_- (PLC_β2_) and inositol 1,4,5 trisphosphate- (IP_3_) dependent release of Ca^2+^ from intracellular stores [97] (Figure 1). In addition to mobilizing intracellular Ca^2+^, G protein-dependent signaling of α7 nAChR can also lead to an activation of the RhoA GTPase, which inhibits neurite growth [98]. In the airways, we have shown that the activation of α3β4 nAChR leads to a release of Ca^2+^ from intracellular stores, resulting in an activation of the TMEM16A chloride channel [5]. This increase in the intracellular Ca^2+^ levels has additional effects on soluble adenylyl cyclases, leading to a rise of intracellular cAMP and protein kinase A activation [5,37] (Figure 1). However, the mechanism that augments the IP_3_ receptor-dependent increase in intracellular Ca^2+^ remains unknown. Based on the existing literature, there might be two possibilities: either the α3β4 nAChR could be coupled to G proteins, as shown for neuronal α7 nAChR in the hippocampus, prefrontal cortex, and striatum [97], or they could be coupled to β-arrestin1, the tyrosine kinase Src and 14-3-3η leading to PLC activation, as it has been recently shown for α4β2 nAChR in transfected HEK293 cells [99]. In line with this, in mitochondria isolated from the squamous lung cell carcinoma line SW900, the α7 nAChR subunit was found to be associated with the phosphoinositide 3-kinase (PI3K) and the β4 subunit with Src [100].

Additionally, the association of α7 nAChR with Janus kinase 2 (JAK2) has been described, and this association leads to activation of the signal transducer and activator of transcription 3 (STAT3), resulting in a localization of STAT3 to the nucleus in neuronal mypHoA-POMC/GFP cells, where it then serves as transcription factor [101] (Figure 1). However, α7 is not the only nAChR coupled to JAK2. Additionally, α4β2 nAChR in stably transfected SH-EP1 neuroblastoma cells are coupled to the JAK2/STAT3 pathway, thereby inhibiting the transcription factor NF_κ_B [102] (Figure 1). Although some of the metabotropic signaling mechanisms of nAChR have only been described in neuronal cells, it is likely that the non-neuronal nAChR of the respiratory tract might also signal via these pathways and might be coupled to G proteins, β-arrestin, JAK2, or Src.

## 9. Possible Roles of Auxiliary Proteins for Nicotinic Receptors

Besides the wide variety of nAChR subtypes resulting in the diverse functions of the receptors, auxiliary proteins, especially of the Ly6/uPAR family, have been found to be able to modulate nAChR function in the respiratory tract. Changes in Lynx1 expression levels were detected in the developing lungs of rhesus monkeys from day 71 of gestation to 12.5 years of age. Lynx1 was predominantly expressed in the airway epithelial cells, submucosal glands, smooth muscle cells, endothelial cells, and alveolar type II cells [103]. In primary bronchial epithelial cell cultures from rhesus macaques, Lynx1 formed a complex with α7 nAChR and led to the inhibition of nAChR-dependent signaling [104]. Due to the negative regulation of Lynx1 on nAChR activity, especially through the inhibition of GABAergic-mediated mucin upregulation, Lynx1-mimetics have been suggested as potential treatment options in asthma and COPD [105]. Additionally, Lynx1 may have beneficial effects in preventing lung cancer endogenously since the increased expression of Lynx1 in A549 lung cancer cells decreased the proliferation, and the inverse knockdown of Lynx1 in these cells led to an increased proliferation [105]. The mechanism of action of Lynx1 on α7 nAChR in A549 cells involved PKC/IP3 signaling as well as the MAP/ERK, p38, and JNK pathways, which then resulted in cell cycle arrest and apoptosis [106]. Supportively, in squamous lung cell carcinomas, Lynx1 expression was much lower than in healthy lung tissue [105].

Another example for a modulatory protein expressed in the respiratory tract, especially in ciliated bronchial epithelial cells of mice, is SLURP-1 [107]. In an asthmatic mouse model, SLURP-1 expression was downregulated, suggesting a protective role for SLURP-1 in preventing airway remodeling under proinflammatory conditions via the upregulation of α7 nAChR activity [108]. In cultured human bronchial epithelial cells, SLURP-1 suppressed the production of the proinflammatory cytokines IL-6 and TNF-α [109]. Interestingly, SLURP-1 seems to have protective effects in lung cancer, as it abolishes the proliferation of A549 cells, prevents the downregulation of PTEN expression, an important tumor suppressor, as well as the up-regulation of α7 nAChR expression [110]. Besides interacting with α7 nAChR, SLURP-1 also forms complexes with the receptor tyrosine kinases EGFR and PRDFRα in A549 cells [111]. The interactions of SLURP-1 with these three proteins down-regulates the migration of A549 cells.

Conclusively, members of the Ly6/uPAR family such as Lnyx1 or SLURP-1, including their synthetic analogues, are promising options for therapeutically targeting lung cancer or asthma.

## 10. Therapeutical Implications for Nicotinic Receptors in the Airways

The various subunits detected in the respiratory tract and especially in the airway epithelium results in the combination of a large variety of nAChR subtypes with different ligand affinities [1]. These subtypes have different functions, as previously described in this review. Thus, selectively targeting these subtypes in the respiratory tract by inhalative medicines or by cell targeted therapies for specific cell types could be beneficial. Until now, anticholinergics targeting muscarinic acetylcholine receptors are widely used for treating asthma and COPD [112]. However, while promising in vivo experiments for modulating nAChR in the respiratory tract have been performed in mouse studies, as summarized in this review, clinical studies in humans are still missing. For example, PNU-282987 has emerged as a potential candidate for modulating α7 nAChR, as convincingly demonstrated in mice [56]. Additionally, GTS-21 could be another candidate for targeting α7 nAChR, as it showed promising effects on peripheral blood mononuclear cells isolated from samples of COPD patients and healthy controls [46]. However, targeting nAChR in the respiratory tract is not trivial because potential candidates may exclusively work in the respiratory tract and not in other organs, so they need to be suitable for inhalation. It should also be considered that in an ideal situation, the nAChR on the apical cytoplasmic membrane may be available for inhalative therapies. The specific targeting of basolateral or mitochondrial nAChR would be even more challenging since the compounds need to be membrane permeable in order to access the receptors at the desired location, but at the end, this might also lead to unfavorable systemic effects. Furthermore, when screening for potential targeting candidates, different stoichiometries of the nAChR subtypes have to be taken into account. So far, nAChR, especially the α7 nAChR subtype, are being targeted pharmaceutically to treat diseases such as schizophrenia and autism in clinical trials [113,114,115,116]. While specific agonists and antagonists exist for α7 nAChR, it could be of considerable interest to develop specific modulators for the other nAChR subtypes that are potentially of therapeutical interest in the respiratory tract, such as the α3β4 or α5 nAChR. Another difficulty is the need for cell type specific therapies, as the α7 nAChR in lung cancer cells should be inhibited, while their activation in alveolar macrophages and epithelial cells could be useful in order for them to exert their anti-inflammatory properties. Despite the obstacles that have yet to be overcome, nAChR represent promising drug targets for airway disease therapies, as shown by encouraging studies in mice [46,56].

## 11. Conclusions

**Figure 2:**
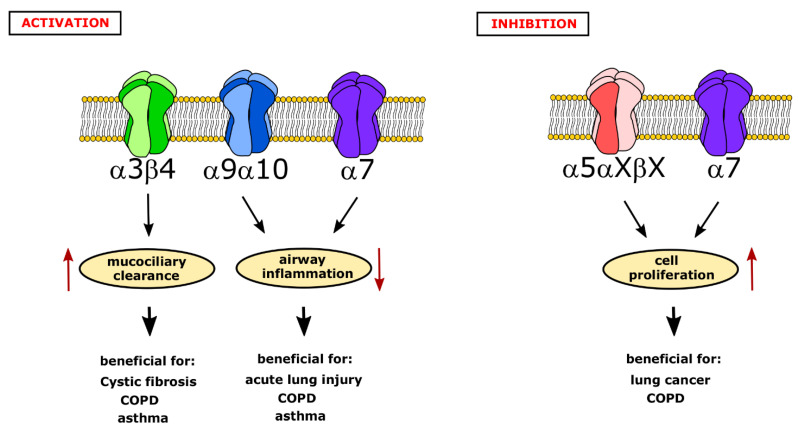
Possible strategies to pharmaceutically target nicotinic acetylcholine receptors (nAChR) in airway diseases. Activation of nAChR has two main beneficial effects: an increase in mucociliary clearance via α3β4 receptors in the epithelium and a decrease of airway inflammation via α9α10 and α7 nAChR present in immune cells. These effects can be useful to attenuate symptoms of various airway diseases, such as cystic fibrosis, chronic obstructive pulmonary disease (COPD), asthma, and acute lung injury. In contrast, the inhibition of α5 nAChR and α7 nAChR could reduce the excess proliferation of airway smooth muscle or cancer cells and therefore could be a suitable therapeutic target for lung cancer or COPD.

Taken together, nAChR play an important role in physiological processes as well as in diseases in the respiratory tract. They are an interesting pharmaceutical target for alternative therapies. However, which receptor subtype should be selectively activated or inhibited needs to be carefully elucidated depending on the disease-specific circumstances. The activation of the α3β4 nAChR could be beneficial in diseases with impaired mucociliary clearance, such as COPD, asthma, or even cystic fibrosis (Figure 2), while the activation of the α9α10 nAChR in immune cells or α7 nAChR in alveolar macrophages and lung epithelial cells might be helpful to ameliorate acute lung injury, COPD, and asthma by reducing airway inflammation (Figure 2). In contrast to this, α5 nAChR should be inhibited rather than activated in order to reduce the cell proliferation of non-small cell lung cancer cells or of airway smooth muscle cells in COPD (Figure 2). The inhibition the α7 nAChR might also be useful in these conditions in tumor cells (Figure 2). Thus, the targeted activation of the α3β4 nAChR exclusively in the respiratory tract—due to its high expression also in the brain—and the inhibition of the α5 nAChR specifically in lung tumors could be interesting therapeutic strategies to follow. The targeting the α7 nAChR should be considered cautiously, as it might have beneficial effects, e.g., the reduction of inflammation upon activation, or worsening effects, e.g., increased cell proliferation. Overall, the development of cell-targeted therapies in recent years, e.g., for non-small-cell lung cancer [117], also presents a large amount of hope for other diseases of the respiratory tract. More preclinical and clinical studies are needed to elucidate the broad impact of nAChR in the respiratory system.

## Data Availability

Not applicable.

## References

[1] Albuquerque E.X., Pereira E.F.R., Alkondon M., Rogers S.W. (2009). Mammalian Nicotinic Acetylcholine Receptors: From Structure to Function. Physiol. Rev..

[2] Razani-Boroujerdi S., Boyd R.T., Dávila-García M.I., Nandi J.S., Mishra N.C., Singh S.P., Pena-Philippides J.C., Langley R., Sopori M.L. (2007). T Cells Express α7-Nicotinic Acetylcholine Receptor Subunits That Require a Functional TCR and Leukocyte-Specific Protein Tyrosine Kinase for Nicotine-Induced Ca 2+ Response. J. Immunol..

[3] Kummer W., Lips K.S., Pfeil U. (2008). The epithelial cholinergic system of the airways. Histochem. Cell Biol..

[4] Hollenhorst M.I., Lips K.S., Weitz A., Krasteva G., Kummer W., Fronius M. (2012). Evidence for functional atypical nicotinic receptors that activate K+-dependent Cl- secretion in mouse tracheal epithelium. Am. J. Respir. Cell Mol. Biol..

[5] Kumar P., Scholze P., Fronius M., Krasteva-Christ G., Hollenhorst M.I. (2020). Nicotine stimulates ion transport via metabotropic b4 subunit containing nicotinic acetylcholine receptors. Br. J. Pharmacol..

[6] Gahring L.C., Myers E.J., Dunn D.M., Weiss R.B., Rogers S.W. (2017). Nicotinic alpha 7 receptor expression and modulation of the lung epithelial response to lipoplysaccharide. PLoS ONE.

[7] Reynolds P.R., Hoidal J.R. (2005). Temporal-spatial expression and transcriptional regulation of α7 nicotinic acetylcholine receptor by thyroid transcription factor-1 and early growth response factor-1 during murine lung development. J. Biol. Chem..

[8] Reynolds P.R., Allison C.H., Willnauer C.P. (2010). TTF-1 regulates α5nicotinic acetylcholine receptor (nAChR) subunits in proximal and distal lung epithelium. Respir. Res..

[9] Fu X.W., Lindstrom J., Spindel E.R. (2009). Nicotine activates and up-regulates nicotinic acetylcholine receptors in bronchial epithelial cells. Am. J. Respir. Cell Mol. Biol..

[10] Maus A.D.J., Pereira E.F.R., Karachunski P.I., Horton R.M., Navaneetham D., Macklin K., Cortes W.S., Albuquerque E.X., Conti-Fine B.M. (1998). Human and rodent bronchial epithelial cells express functional nicotinic acetylcholine receptors. Mol. Pharmacol..

[11] Wang Y., Pereira E.F.R., Maus A.D.J., Ostlie N.S., Navaneetham D., Lei S., Albuquerque E.X., Conti-Fine B.M. (2001). Human bronchial epithelial and endothelial cells express α7 nicotinic acetylcholine receptors. Mol. Pharmacol..

[12] Diabasana Z., Perotin J., Belgacemi R., Ancel J., Polette M., Desl G., Dormoy V. (2020). Nicotinic Receptor Subunits Atlas in the Adult. Int. J. Mol. Sci..

[13] Mikulski Z., Hartmann P., Jositsch G., Zasłona Z., Lips K.S., Pfeil U., Kurzen H., Lohmeyer J., Clauss W.G., Grau V. (2010). Nicotinic receptors on rat alveolar macrophages dampen ATP-induced increase in cytosolic calcium concentration. Respir. Res..

[14] Lykhmus O., Gergalova G., Koval L., Zhmak M., Komisarenko S., Skok M. (2014). Mitochondria express several nicotinic acetylcholine receptor subtypes to control various pathways of apoptosis induction. Int. J. Biochem. Cell Biol..

[15] Jositsch G., Papadakis T., Haberberger R.V., Wolff M., Wess J., Kummer W. (2009). Suitability of muscarinic acetylcholine receptor antibodies for immunohistochemistry evaluated on tissue sections of receptor gene-deficient mice. Naunyn. Schmiedebergs. Arch. Pharmacol..

[16] Moser N., Mechawar N., Jones I., Gochberg-Sarver A., Orr-Urtreger A., Plomann M., Salas R., Molles B., Marubio L., Roth U. (2007). Evaluating the suitability of nicotinic acetylcholine receptor antibodies for standard immunodetection procedures. J. Neurochem..

[17] Chernyavsky A., Chen Y., Wang P.H., Grando S.A. (2015). Pemphigus vulgaris antibodies target the mitochondrial nicotinic acetylcholine receptors that protect keratinocytes from apoptolysis. Int. Immunopharmacol..

[18] Lam D.C.L., Luo S.Y., Fu K.H., Lui M.M.S., Chan K.H., Wistuba I.I., Gao B., Tsao S.W., Ip M.S.M., Minna J.D. (2016). Nicotinic acetylcholine receptor expression in human airway correlates with lung function. Am. J. Physiol.—Lung Cell. Mol. Physiol..

[19] Nielsen B.E., Minguez T., Bermudez I., Bouzat C. (2018). Molecular function of the novel α7β2 nicotinic receptor. Cell. Mol. Life Sci..

[20] Lustig L.R. (2006). Nicotinic acetylcholine receptor structure and function in the efferent auditory system. Anat. Rec.—Part. A Discov. Mol. Cell. Evol. Biol..

[21] Krashia P., Moroni M., Broadbent S., Hofmann G., Kracun S., Beato M., Groot-Kormelink P.J., Sivilotti L.G. (2010). Human α3β4 neuronal nicotinic receptors show different stoichiometry if they are expressed in Xenopus oocytes or mammalian HEK293 cells. PLoS ONE.

[22] Moroni M., Zwart R., Sher E., Cassles B.K., Bermudez I. (2006). 4beta2 Nicotinic Receptors with High and Low Acetylcholine Sensitivity: Pharmacology, Stoichiometry, and Sensitivity to Long-Term Exposure to Nicotine. Mol. Pharmacol..

[23] Lamotte d’Incamps B., Zorbaz T., Dingova D., Krejci E., Ascher P. (2018). Stoichiometry of the heteromeric nicotinic receptors of the renshaw cell. J. Neurosci..

[24] Scholze P., Huck S. (2020). The α5 Nicotinic Acetylcholine Receptor Subunit Differentially Modulates α4β2* and α3β4* Receptors. Front. Synaptic Neurosci..

[25] Audrit K.J., Delventhal L., Aydin Ö., Nassenstein C. (2017). The nervous system of airways and its remodeling in inflammatory lung diseases. Cell Tissue Res..

[26] Krasteva G., Canning B.J., Hartmann P., Veres T.Z., Papadakis T., Muhlfeld C., Schliecker K., Tallini Y.N., Braun A., Hackstein H. (2011). Cholinergic chemosensory cells in the trachea regulate breathing. Proc. Natl. Acad. Sci. USA.

[27] Hollenhorst M.I., Jurastow I., Nandigama R., Appenzeller S., Li L., Vogel J., Wiederhold S., Althaus M., Empting M., Altmüller J. (2020). Tracheal brush cells release acetylcholine in response to bitter tastants for paracrine and autocrine signaling. FASEB J..

[28] Perniss A., Liu S., Boonen B., Keshavarz M., Ruppert A.L., Timm T., Pfeil U., Soultanova A., Kusumakshi S., Delventhal L. (2020). Chemosensory Cell-Derived Acetylcholine Drives Tracheal Mucociliary Clearance in Response to Virulence-Associated Formyl Peptides. Immunity.

[29] Saunders C.J., Christensen M., Finger T.E., Tizzano M. (2014). Cholinergic neurotransmission links solitary chemosensory cells to nasal inflammation. Proc. Natl. Acad. Sci. USA.

[30] Bakshani C.R., Morales-Garcia A.L., Althaus M., Wilcox M.D., Pearson J.P., Bythell J.C., Burgess J.G. (2018). Evolutionary conservation of the antimicrobial function of mucus: A first defence against infection. npj Biofilms Microbiomes.

[31] Klein M.K., Haberberger R.V., Hartmann P., Faulhammer P., Lips K.S., Krain B., Wess J., Kummer W., König P. (2009). Muscarinic receptor subtypes in cilia-driven transport and airway epithelial development. Eur. Respir. J..

[32] Zagoory O., Braiman A., Priel Z. (2002). The mechanism of ciliary stimulation by acetylcholine: Roles of calcium, PKA, and PKG. J. Gen. Physiol..

[33] Schmid A., Salathe M. (2011). Ciliary beat co-ordination by calcium. Biol. Cell.

[34] Acevedo M. (1994). Effects of acetyl choline on ion transport in sheep tracheal epithelium. Pflügers Arch. Eur. J. Physiol..

[35] Hollenhorst M.I., Lips K.S., Wolff M., Wess J., Gerbig S., Takats Z., Kummer W., Fronius M. (2012). Luminal cholinergic signalling in airway lining fluid: A novel mechanism for activating chloride secretion via Ca^2+^ -dependent Cl^−^ and K^+^ channels. Br. J. Pharmacol..

[36] Dittrich N.P., Kummer W., Clauss W.G., Fronius M. (2015). Luminal acetylcholine does not affect the activity of the CFTR in tracheal epithelia of pigs. Int. Immunopharmacol..

[37] Hollenhorst M.I., Lips K.S., Kummer W., Fronius M. (2012). Nicotine-induced activation of soluble adenylyl cyclase participates in ion transport regulation in mouse tracheal epithelium. Life Sci..

[38] Perniss A., Latz A., Boseva I., Papadakis T., Dames C., Meisel C., Meisel A., Scholze P., Kummer W., Krasteva-Christ G. (2020). Acute nicotine administration stimulates ciliary activity via α3β4 nAChR in the mouse trachea. Int. Immunopharmacol..

[39] Blank U., Rückes C., Clauss W., Weber W.M. (1997). Effects of nicotine on human nasal epithelium: Evidence for nicotinic receptors in non-excitable cells. Pflugers Arch. Eur. J. Physiol..

[40] Maouche K., Medjber K., Zahm J.-M., Delavoie F., Terryn C., Coraux C., Pons S., Cloez-Tayarani I., Maskos U., Birembaut P. (2013). Contribution of 7 nicotinic receptor to airway epithelium dysfunction under nicotine exposure. Proc. Natl. Acad. Sci. USA.

[41] Kistemaker L.E.M., Gosens R. (2015). Acetylcholine beyond bronchoconstriction: Roles in inflammation and remodeling. Trends Pharmacol. Sci..

[42] Belmonte K.E. (2005). Cholinergic pathways in the lungs and anticholinergic therapy for chronic obstructive pulmonary disease. Proc. Am. Thorac. Soc..

[43] Gosens R., Zaagsma J., Meurs H., Halayko A.J. (2006). Muscarinic receptor signaling in the pathophysiology of asthma and COPD. Respir. Res..

[44] Wang H., Yu M., Ochani M., Amella C.A., Tanovic M., Susarla S., Li J.H., Wang H., Yang H., Ulloa L. (2003). Nicotinic acetylcholine receptor α7 subunit is an essential regulator of inflammation. Nature.

[45] Zhu S., Huang S., Xia G., Wu J., Shen Y., Wang Y., Ostrom R.S., Du A., Shen C., Xu C. (2021). Anti-inflammatory effects of α7-nicotinic ACh receptors are exerted through interactions with adenylyl cyclase-6. Br. J. Pharmacol..

[46] Douaoui S., Djidjik R., Boubakeur M., Ghernaout M., Touil-boukoffa C., Oumouna M., Derrar F., Amrani Y. (2020). GTS-21, an α 7nAChR agonist, suppressed the production of key in fl ammatory mediators by PBMCs that are elevated in COPD patients and associated with impaired lung function. Immunobiology.

[47] Sitapara R.A., Gauthier A.G., Valdés-Ferrer S.I., Lin M., Patel V., Wang M., Martino A.T., Perron J.C., Ashby C.R., Tracey K.J. (2020). The α7 nicotinic acetylcholine receptor agonist, GTS-21, attenuates hyperoxia-induced acute inflammatory lung injury by alleviating the accumulation of HMGB1 in the airways and the circulation. Mol. Med..

[48] Yan F., Gao H., Zhao H., Bhatia M., Zeng Y. (2018). Roles of airway smooth muscle dysfunction in chronic obstructive pulmonary disease. J. Transl. Med..

[49] Hong W., Peng G., Hao B., Liao B., Zhao Z., Zhou Y., Peng F., Ye X., Huang L., Zheng M. (2017). Nicotine-Induced Airway Smooth Muscle Cell Proliferation Involves TRPC6-Dependent Calcium Influx Via α7 nAChR. Cell. Physiol. Biochem..

[50] Jiang Y., Zhou Y., Peng G., Tian H., Pan D., Liu L., Yang X., Li C., Li W., Chen L. (2019). TRPC channels mediated calcium entry is required for proliferation of human airway smooth muscle cells induced by nicotine-nAChR. Biochimie.

[51] Blanchet M.R., Israël-Assayag E., Cormier Y. (2005). Modulation of airway inflammation and resistance in mice by a nicotinic receptor agonist. Eur. Respir. J..

[52] Gahring L.C., Myers E.J., Dunn D.M., Weiss R.B., Rogers S.W. (2018). Lung eosinophilia induced by house dust mites or ovalbumin is modulated by nicotinic receptor α7 and inhibited by cigarette smoke. Am. J. Physiol.—Lung Cell. Mol. Physiol..

[53] Zheng H., Zhang Y., Pan J., Liu N., Qin Y., Qiu L., Liu M., Wang T. (2021). The Role of Type 2 Innate Lymphoid Cells in Allergic Diseases. Front. Immunol..

[54] Feng X., Li L., Feng J., He W., Li N., Shi T., Jie Z., Su X. (2021). Vagal-α7nAChR signaling attenuates allergic asthma responses and facilitates asthma tolerance by regulating inflammatory group 2 innate lymphoid cells. Immunol. Cell Biol..

[55] Galle-Treger L., Suzuki Y., Patel N., Sankaranarayanan I., Aron J.L., Maazi H., Chen L., Akbari O. (2016). Nicotinic acetylcholine receptor agonist attenuates ILC2-dependent airway hyperreactivity. Nat. Commun..

[56] Yuan F., Jiang L., Li Q., Sokulsky L., Wanyan Y., Wang L., Liu X., Zhou L., Tay H.L., Zhang G. (2021). A Selective α7 Nicotinic Acetylcholine Receptor Agonist, PNU-282987, Attenuates ILC2s Activation and Alternaria-Induced Airway Inflammation. Front. Immunol..

[57] Bankova L.G., Dwyer D.F., Yoshimoto E., Ualiyeva S., McGinty J.W., Raff H., von Moltke J., Kanaoka Y., Frank Austen K., Barrett N.A. (2018). The cysteinyl leukotriene 3 receptor regulates expansion of IL-25–producing airway brush cells leading to type 2 inflammation. Sci. Immunol..

[58] Brégeon F., Xeridat F., Andreotti N., Lepidi H., Delpierre S., Roch A., Ravailhe S., Jammes Y., Steinberg J.G. (2011). Activation of nicotinic cholinergic receptors prevents ventilator-induced lung injury in rats. PLoS ONE.

[59] Dos Santos C.C., Shan Y., Akram A., Slutsky A.S., Haitsma J.J. (2011). Neuroimmune regulation of ventilator-induced lung injury. Am. J. Respir. Crit. Care Med..

[60] Su X., Jae W.L., Matthay Z.A., Mednick G., Uchida T., Fang X., Gupta N., Matthay M.A. (2007). Activation of the α7 nAChR reduces acid-induced acute lung injury in mice and rats. Am. J. Respir. Cell Mol. Biol..

[61] Vicary G.W., Ritzenthaler J.D., Panchabhai T.S., Torres-González E., Roman J. (2017). Nicotine stimulates collagen type I expression in lung via α7 nicotinic acetylcholine receptors. Respir. Res..

[62] Tao M., Liu Q., Miyazaki Y., Canning B.J. (2019). Nicotinic receptor dependent regulation of cough and other airway defensive reflexes. Pulm. Pharmacol. Ther..

[63] de Jonge W.J., van der Zanden E.P., The F.O., Bijlsma M.F., van Westerloo D.J., Bennink R.J., Berthoud H.R., Uematsu S., Akira S., van den Wijngaard R.M. (2005). Stimulation of the vagus nerve attenuates macrophage activation by activating the Jak2-STAT3 signaling pathway. Nat. Immunol..

[64] Wang J., Li R., Peng Z., Zhou W., Hu B., Rao X., Yang X., Li J. (2019). GTS-21 Reduces Inflammation in Acute Lung Injury by Regulating M1 Polarization and Function of Alveolar Macrophages. Shock.

[65] Pinheiro N.M., Santana F.P.R., Almeida R.R., Guerreiro M., Martins M.A., Caperuto L.C., Câmara N.O.S., Wensing L.A., Prado V.F., Tiberio I.F.L.C. (2017). Acute lung injury is reduced by the a7nAChR agonist PNU-282987 through changes in the macrophage profile. FASEB J..

[66] Rodero M.P., Poupel L., Loyher P.L., Hamon P., Licata F., Pessel C., Hume D.A., Combadière C., Boissonnas A. (2015). Immune surveillance of the lung by migrating tissue monocytes. Elife.

[67] Van Der Zanden E.P., Hilbers F.W., Verseijden C., Van Den Wijngaard R.M., Skynner M., Lee K., Ulloa L., Boeckxstaens G.E., De Jonge W.J. (2012). Nicotinic acetylcholine receptor expression and susceptibility to cholinergic immunomodulation in human monocytes of smoking individuals. Neuroimmunomodulation.

[68] Richter K., Mathes V., Fronius M., Althaus M., Hecker A., Krasteva-Christ G., Padberg W., Hone A.J., McIntosh J.M., Zakrzewicz A. (2016). Phosphocholine-an agonist of metabotropic but not of ionotropic functions of α9-containing nicotinic acetylcholine receptors. Sci. Rep..

[69] Zakrzewicz A., Richter K., Agné A., Wilker S., Siebers K., Fink B., Krasteva-Christ G., Althaus M., Padberg W., Hone A.J. (2017). Canonical and Novel Non-Canonical Cholinergic Agonists Inhibit ATP-Induced Release of Monocytic Interleukin-1β via Different Combinations of Nicotinic Acetylcholine Receptor Subunits α7, α9 and α10. Front. Cell. Neurosci..

[70] Richter K., Sagawe S., Hecker A., Küllmar M., Askevold I., Damm J., Heldmann S., Pöhlmann M., Ruhrmann S., Sander M. (2018). C-Reactive Protein Stimulates Nicotinic Acetylcholine Receptors to Control ATP-Mediated Monocytic Inflammasome Activation. Front. Immunol..

[71] Siebers K., Fink B., Zakrzewicz A., Agné A., Richter K., Konzok S., Hecker A., Zukunft S., Küllmar M., Klein J. (2018). Alpha-1 Antitrypsin Inhibits ATP-Mediated Release of Interleukin-1β via CD36 and Nicotinic Acetylcholine Receptors. Front. Immunol..

[72] Richter K., Koch C., Perniss A., Wolf P., Schweda E., Wichmann S., Wilker S., Magel I., Sander M., McIntosh J. (2018). Phosphocholine-Modified Lipooligosaccharides of Haemophilus influenzae Inhibit ATP-Induced IL-1β Release by Pulmonary Epithelial Cells. Molecules.

[73] Backhaus S., Zakrzewicz A., Richter K., Damm J., Wilker S., Fuchs-Moll G., Küllmar M., Hecker A., Manzini I., Ruppert C. (2017). Surfactant inhibits ATP-induced release of interleukin-1β via nicotinic acetylcholine receptors. J. Lipid Res..

[74] Richter K., Ogiemwonyi-Schaefer R., Wilker S., Chaveiro A.I., Agné A., Hecker M., Reichert M., Amati A.-L., Schlüter K.-D., Manzini I. (2020). Amyloid Beta Peptide (Aβ1-42) Reverses the Cholinergic Control of Monocytic IL-1β Release. J. Clin. Med..

[75] Valdez-Miramontes C.E., Trejo Martínez L.A., Torres-Juárez F., Rodríguez Carlos A., Marin-Luévano S.P., de Haro-Acosta J.P., Enciso-Moreno J.A., Rivas-Santiago B. (2020). Nicotine modulates molecules of the innate immune response in epithelial cells and macrophages during infection with M. tuberculosis. Clin. Exp. Immunol..

[76] Rosas-Ballina M., Goldstein R.S., Gallowitsch-Puerta M., Yang L., Valdés-Ferrer S.I., Patel N.B., Chavan S., Al-Abed Y., Yang H., Tracey K.J. (2009). The selective α7 agonist GTS-21 attenuates cytokine production in human whole blood and human monocytes activated by ligands for TLR2, TLR3, TLR4, TLR9, and RAGE. Mol. Med..

[77] Miramontes C.V., Rodríguez-Carlos A., Marin-Luévano S.P., Trejo Martínez L.A., de Haro Acosta J., Enciso-Moreno J.A., Rivas-Santiago B. (2020). Nicotine promotes the intracellular growth of Mycobacterium tuberculosis in epithelial cells. Tuberculosis.

[78] Lupacchini L., Maggi F., Tomino C., De Dominicis C., Mollinari C., Fini M., Bonassi S., Merlo D., Russo P. (2021). Nicotine Changes Airway Epithelial Phenotype and May Increase the SARS-COV-2 Infection Severity. Molecules.

[79] Hoffmann M., Kleine-Weber H., Schroeder S., Krüger N., Herrler T., Erichsen S., Schiergens T.S., Herrler G., Wu N.H., Nitsche A. (2020). SARS-CoV-2 Cell Entry Depends on ACE2 and TMPRSS2 and Is Blocked by a Clinically Proven Protease Inhibitor. Cell.

[80] Miyara M., Tubach F., POURCHER V., Morelot-Panzini C., Pernet J., Haroche J., Lebbah S., Morawiec E., Gorochov G., Caumes E. (2020). Low rate of daily active tobacco smoking in patients with symptomatic COVID-19. Qeios.

[81] Tizabi Y., Getachew B., Copeland R.L., Aschner M. (2020). Nicotine and the nicotinic cholinergic system in COVID-19. FEBS J..

[82] Korzeniowska A., Reka G., Bilsaka M., Piecewicz-Szczesna H. (2021). The Smoker’s papdox during the COVID-19 pandemic? The influence of smoking and vaping on the incidence and course of SARS-CoV-2 virus infection as well as possibility of using nicotine in the treatment of COVID-19–Review of the literature. Epidemiol. Rev..

[83] Wang S., Hu Y. (2018). α7 nicotinic acetylcholine receptors in lung cancer (Review). Oncol. Lett..

[84] Dasgupta P., Rastogi S., Pillai S., Ordonez-Ercan D., Morris M., Haura E., Chellappan S. (2006). Nicotine induces cell proliferation by β-arrestin-mediated activation of Src and Rb-Raf-1 pathways. J. Clin. Invest..

[85] Schaal C., Chellappan S. (2016). Nicotine-mediated regulation of nicotinic acetylcholine receptors in non-small cell lung adenocarcinoma by E2F1 and STAT1 transcription factors. PLoS ONE.

[86] Zhang C., Ding X.P., Zhao Q.N., Yang X.J., An S.M., Wang H., Xu L., Zhu L., Chen H.Z. (2016). Role of a7-nicotinic acetylcholine receptor in nicotine-induced invasion and epithelial-to-mesenchymal transition in human non-small cell lung cancer cells. Oncotarget.

[87] Zhang C., Yu P., Zhu L., Zhao Q., Lu X., Bo S. (2017). Blockade of α7 nicotinic acetylcholine receptors inhibit nicotine-induced tumor growth and vimentin expression in non-small cell lung cancer through MEK/ERK signaling way. Oncol. Rep..

[88] Mucchietto V., Fasoli F., Pucci S., Moretti M., Benfante R., Maroli A., Di Lascio S., Bolchi C., Pallavicini M., Dowell C. (2018). α9- and α7-containing receptors mediate the pro-proliferative effects of nicotine in the A549 adenocarcinoma cell line. Br. J. Pharmacol..

[89] Chikova A., Grando S.A. (2011). Naturally occurring variants of human A9 nicotinic receptor differentially affect bronchial cell proliferation and transformation. PLoS ONE.

[90] Improgo M.R.D., Scofield M.D., Tapper A.R., Gardner P.D. (2010). The nicotinic acetylcholine receptor CHRNA5/A3/B4 gene cluster: Dual role in nicotine addiction and lung cancer. Prog. Neurobiol..

[91] Ma X., Jia Y., Zu S., Li R., Jia Y., Zhao Y., Xiao D., Dang N., Wang Y. (2014). α5 Nicotinic acetylcholine receptor mediates nicotine-induced HIF-1α and VEGF expression in non-small cell lung cancer. Toxicol. Appl. Pharmacol..

[92] Sun H., Ma X. (2015). α5-nAChR modulates nicotine-induced cell migration and invasion in A549 lung cancer cells. Exp. Toxicol. Pathol..

[93] Sun H.J., Jia Y.F., Ma X.L. (2017). Alpha5 nicotinic acetylcholine receptor contributes to nicotine-induced lung cancer development and progression. Front. Pharmacol..

[94] Zhang Y., Jia Y., Li P., Li H., Xiao D., Wang Y., Ma X. (2017). Reciprocal activation of α5-nAChR and STAT3 in nicotine-induced human lung cancer cell proliferation. J. Genet. Genomics.

[95] Chen X., Jia Y., Zhang Y., Zhou D., Sun H., Ma X. (2020). α5-nAChR contributes to epithelial-mesenchymal transition and metastasis by regulating Jab1/Csn5 signalling in lung cancer. J. Cell. Mol. Med..

[96] Chernyavsky A.I., Shchepotin I.B., Galitovkiy V., Grando S.A. (2015). Mechanisms of tumor-promoting activities of nicotine in lung cancer: Synergistic effects of cell membrane and mitochondrial nicotinic acetylcholine receptors. BMC Cancer.

[97] King J.R., Nordman J.C., Bridges S.P., Lin M.K., Kabbani N. (2015). Identification and characterization of a G protein-binding cluster in α7 nicotinic acetylcholine receptors. J. Biol. Chem..

[98] King J.R., Kabbani N. (2016). Alpha 7 nicotinic receptor coupling to heterotrimeric G proteins modulates RhoA activation, cytoskeletal motility, and structural growth. J. Neurochem..

[99] Acharya S., Kundu D., Choi H.J., Kim K.M. (2020). Metabotropic signaling cascade involved in α4β2 nicotinic acetylcholine receptor-mediated PKCβII activation. Biochim. Biophys. Acta—Mol. Cell Res..

[100] Chernyavsky A.I., Shchepotin I.B., Grando S.A. (2015). Mechanisms of growth-promoting and tumor-protecting effects of epithelial nicotinic acetylcholine receptors. Int. Immunopharmacol..

[101] Souza C.M., Libardi do Amaral C., Souza S.C., Parras de Souza A.C., de Cassia Alves Martins I., Contieri L.S., Milanski M., Torsoni A.S., Ignacio-Souza L.M., Torsoni M.A. (2019). JAK2/STAT3 Pathway is Required for α7nAChR-Dependent Expression of POMC and AGRP Neuropeptides in Male Mice. Cell. Physiol. Biochem..

[102] Hosur V., Loring R.H. (2011). a4b2 Nicotinic Receptors Partially Mediate Anti-Inflammatory Effects through Janus Kinase 2-Signal Transducer and Activator of Transcription 3 but Not Calcium or cAMP Signaling. Mol. Pharmacol..

[103] Sekhon H.S., Song P., Jia Y., Lindstrom J., Spindel E.R. (2005). Expression of lynx1 in developing lung and its modulation by prenatal nicotine exposure. Cell Tissue Res..

[104] Fu X.W., Rekow S.S., Spindel E.R. (2012). The ly-6 protein, lynx1, is an endogenous inhibitor of nicotinic signaling in airway epithelium. Am. J. Physiol.—Lung Cell. Mol. Physiol..

[105] Fu X.W., Song P.F., Spindel E.R. (2015). Role of Lynx1 and related Ly6 proteins as modulators of cholinergic signaling in normal and neoplastic bronchial epithelium. Int. Immunopharmacol..

[106] Bychkov M., Shenkarev Z., Shulepko M., Shlepova O., Kirpichnikov M., Lyukmanova E. (2019). Water-soluble variant of human Lynx1 induces cell cycle arrest and apoptosis in lung cancer cells via modulation of α7 nicotinic acetylcholine receptors. PLoS ONE.

[107] Horiguchi K., Horiguchi S., Yamashita N., Irie K., Masuda J., Takano-Ohmuro H., Himi T., Miyazawa M., Moriwaki Y., Okuda T. (2009). Expression of SLURP-1, an endogenous α7 nicotinic acetylcholine receptor allosteric ligand, in murine bronchial epithelial cells. J. Neurosci. Res..

[108] Narumoto O., Horiguchi K., Horiguchi S., Moriwaki Y., Takano-Ohmuro H., Shoji S., Misawa H., Yamashita N., Nagase T., Kawashima K. (2010). Down-regulation of secreted lymphocyte antigen-6/urokinase-type plasminogen activator receptor-related peptide-1 (SLURP-1), an endogenous allosteric α7 nicotinic acetylcholine receptor modulator, in murine and human asthmatic conditions. Biochem. Biophys. Res. Commun..

[109] Narumoto O., Niikura Y., Ishii S., Morihara H., Okashiro S., Nakahari T., Nakano T., Matsumura H., Shimamoto C., Moriwaki Y. (2013). Effect of secreted lymphocyte antigen-6/urokinase-type plasminogen activator receptor-related peptide-1 (SLURP-1) on airway epithelial cells. Biochem. Biophys. Res. Commun..

[110] Shulepko M.A., Bychkov M.L., Shlepova O.V., Shenkarev Z.O., Kirpichnikov M.P., Lyukmanova E.N. (2020). Human secreted protein SLURP-1 abolishes nicotine-induced proliferation, PTEN down-regulation and α7-nAChR expression up-regulation in lung cancer cells. Int. Immunopharmacol..

[111] Bychkov M.L., Shulepko M.A., Shlepova O.V., Kulbatskii D.S., Chulina I.A., Paramonov A.S., Baidakova L.K., Azev V.N., Koshelev S.G., Kirpichnikov M.P. (2021). SLURP-1 Controls Growth and Migration of Lung Adenocarcinoma Cells, Forming a Complex With α7-nAChR and PDGFR/EGFR Heterodimer. Front. Cell Dev. Biol..

[112] Gosens R., Gross N. (2018). The mode of action of anticholinergics in asthma. Eur. Respir. J..

[113] Olincy A., Blakeley-Smith A., Johnson L., Kem W.R., Freedman R. (2016). Brief Report: Initial Trial of Alpha7-Nicotinic Receptor Stimulation in Two Adult Patients with Autism Spectrum Disorder. J. Autism Dev. Disord..

[114] Lieberman J.A., Dunbar G., Segreti A.C., Girgis R.R., Seoane F., Beaver J.S., Duan N., Hosford D.A. (2013). A randomized exploratory trial of an alpha-7 nicotinic receptor agonist (TC-5619) for cognitive enhancement in schizophrenia. Neuropsychopharmacology.

[115] Antonio-Tolentino K., Hopkins C.R. (2020). Selective α7 nicotinic receptor agonists and positive allosteric modulators for the treatment of schizophrenia–a review. Expert Opin. Investig. Drugs.

[116] Tregellas J.R., Wylie K.P. (2019). Alpha7 nicotinic receptors as therapeutic targets in schizophrenia. Nicotine Tob. Res..

[117] Yuan M., Huang L.L., Chen J.H., Wu J., Xu Q. (2019). The emerging treatment landscape of targeted therapy in non-small-cell lung cancer. Signal. Transduct. Target. Ther..

